# Editorial

**Published:** 1871-08

**Authors:** 


### Editorial.

### *The Massacre of the Innocents.*

"A voice was heard in Rama, lamentation and great mourning: Rachel bewailing her children, and would not be comforted because they are not."

Perhaps no prophecy was ever uttered, by divine inspiration, of more universal applicability than the above. Significant at that day, in the mouth of Jeremiah, of the sacrifice of all in Judea of two years old and under, on the altar of political jealousy, it is not less so to-day of the continued slaughter of the same class upon the altar of human ignorance and stupidity.

This parallel will readily suggest itself to the mind of the thoughtful reader of the sanitary statistics of this and all other large cities, during the present season of the year.

A glance at the mortality reports of our accomplished Sanitary Superintendent from week to week, while showing the most favorable results from the efforts at sanitary reform, will also demonstrate the undiminished ratio of infant mortality.

When, in 1867, epidemic cholera numbered among its victims fifteen hundred of our citizens, not only the whole city but the entire country was panic stricken, and, shrinking in terror from the fearful scourge, spared no pains, no expense, to devise and put into operation means for averting the danger. Science and capital co-operated heartily in the work of self-preservation, and to their united efforts we owe, in part at least, our present immunity from this danger.

When, on the other hand, in 1870, the reports of the registrar of vital statistics of Chicago show a mortality amongst children of nearly eight hundred from "cholera infantum" alone, and of about as many more classified under the various heads, diarrhœa, teething, convulsions, summer complaint, dysentery, etc., no fears are awakened, no special measures are concerted, no funds are contributed, to avert this plague; but, weeping apathetically over the graves of the slaughtered innocents, one and all execrate the "hot weather" or "the teeth," as if children could not live in an atmosphere above 90° Fahrenheit, or dentition was a necessarily fatal ordeal which was only exceptionally survived.

How thoroughly true it is that "familiarity breeds contempt." Accustomed yearly to see hundreds of children withering under this blight, it has come to be considered a matter of course, and the ordinary coincidences, heat and dentition, being assumed as the causes, no further effort is made to detect other and more direct agencies in the production of a pestilence whose only result is the death of a few children every summer. The magnitude of the evil is lost sight of in its continuous presence.

While it is doubtless true that a high atmospheric temperature is a factor, and an essential factor, in the production of cholera infantum, a correct appreciation of the subject will never be acquired without a thorough comprehension of concurrent causes. If solar heat be the sole cause of cholera infantum, then the elevation of temperature to a definite degree would be the signal for a universal epidemic, or if dentition were *the cause*, no child would escape while passing through this phase of its development.

Again, we hear it asserted confidently that the excessive infant mortality from this cause is a result of exposure to foul exhalations from filthy streets, gutters, and alleys, and from the unwholesome atmosphere engendered in the crowded tenements of the poor, but, at the same time, the mortality lists show that cholera infantum is no respecter of localities, and, like its grim general, "pallida mors æquo pulsat pede pauperum tabernas regumque turris." (Hor.)

Since it is thus apparent that neither heat, dentition nor impure air can alone occasion the development of this disease in the infantile organism, and that of these heat is the only constant factor, it follows that some other constant factor or factors must exist, which concurrently with these, with the first invariably, with the other incidentally, operate to produce the diseased condition in question.

What, then, are these other constant factors? Are they susceptible of detection? One of them certainly is so, and that one is Starvation.

It may seem a bold assertion and a libel upon humanity to charge the mothers of our city and of our country with the horrible crime of infanticide, and yet we have no hesitation in asserting that hundreds of children, every year, are starved to death—starved in the midst of plenty—starved through the deplorable ignorance of their parents, and the criminal ignorance of even some physicians.

It would seem to be quite time to understand that the cavity of the stomach, so far as nutrition is concerned, is entirely outside of the organism, and that the filling of this cavity does not necessarily imply the feeding of the organism. Place within this receptacle matters insusceptible of solution in its secretions, and therefore of absorption by its vessels, and the organism is as certainly starved as by the total privation of food.

Now this condition is of daily occurrence within the experience of every physician, and this very frequency of occurrence renders excusable the denunciation of an evil which should be so apparent as to render its denunciation impertinent.

If strict inquiry be made into the history of every case of infantile intestinal disease, one constant antecedent will appear, viz., the ingestion of indigestible matter, and of these, in the vast majority of cases, the offending substance will be found to be starch in some one of its various forms.

From the food provided by the hand of Omniscience for the human infant, this compound has been entirely excluded, which seems to be a sufficient indication of its unfitness for their nutrition; but man, ever seeking to be wiser than his Maker, striving to improve upon the plan of creation, substitutes for the mother's milk, first that of swill-fed cows, and, next, starch, bread, crackers, arrowroot, sago, rice, farina, and so on through the long category of infantile poisons, and succeeds in producing cholera-infantum, and raising the price of cemetery companies' stock.

If some of the medical gentlemen who are in the habit of prescribing, for their infantile patients, from the above dietary list, will take the trouble to examine the alvine evacuations of the little victims with a microscope, they will perceive, to their great astonishment, doubtless, the starch corpuscles unchanged therein, and will, perhaps, ask how is this? We answer, with Pandams, "he that would have a cake out of the wheat must tarry the grinding"; but a faute de moulin point de farine; and here lies the whole secret in a nutshell, wait for the grinding, wait for the grinders, and when they make their appearance then give farinaceous food, and not until then. Pursue this course, and its wisdom will be demonstrated by the diminished ratio of infant mortality, which will surely follow; continue to neglect it, continue to violate physiology, or, what is the same thing, the laws of nature, and we shall continue to see the mortality of our city rise to the appalling figures of last Saturday, viz., 1-60 of one per cent. per diem, *i. e.* at the rate of 6 per cent. per annum, which would reduce the average longevity to 16 years and 8 months.

W. H.

***Cundurango.***

From an analysis of this drug, by Dr. Antisell, of Washington, D. C., published in the July number of the *American Journal of Pharmacy*, its alleged therapeutic activity appears to rest upon a very slender foundation.

It is separable mechanically into wood and bark, in the proportion of 50.28 parts per cent. of the former and 49.72 parts of the latter. Of these, the bark is asserted to contain all the medicinal virtues residing in the plant.

Of this latter, 100 parts yielded of water 8 per cent., of mineral salts (ash) 12, and of vegetable substance 80 per cent.

The 80 per cent. of vegetable matter yielded 63.5 per cent. of cellulose, lignin, etc.; of tannin, yellow and brown coloring matters extractive, 12.6 per cent.; of gum and glucose from starch, .5 of 1 per cent.; of yellow resin, soluble in alcohol, 2.7 per cent.; and of fatty matters soluble in ether, and partially so in strong alcohol, .7 of 1 per cent. No crystalline alkaloid or active principle was detected.

The Doctor summarizes his conclusions thus—"Whatever medicinal virtues the plant may possess, must reside either in the yellow resin or the extractive; the former is soluble in alcohol, the latter in water; in the watery decoction, some of the resin is diffused, but the greater portion of the resin is not extracted by the water. The therapeutic position of the plant, judged from analysis, might be among the aromatic bitters."

We fear that the gentleman of this city who has ordered thirty pounds for experiment, will find that he has an elephant on his hands. W. H.

This is what a respectable medical paper says of the book castigated last February in this Journal by *Volta Reeke*, and over the review of which the hypocrites and noodles waxed virtuously bellicose:

"A Panel House Book.—The mania for sensational books seems at last to have affected medical writers also. Beginning with small volumes bearing an innocent question on the title page, we next had 'Frenchy' translations of French authors. And now the climax seems to have been reached in a work which has been sent us lately. We never met with a scribbler before who could write so perfectly de omnibus rebus et quibusdam aliis. As is always the case in such books, exceptional cases are dragged forth as being the rule; everything and everybody is, in the opinion of the writer, wrong, civilization is wrong, men are brutes, women are depraved, girls and boys are extremely wicked, and even little children, who generally are supposed to be mere angels without wings, are by him accused and condemned. The laws of logic seem to be entirely unknown to the writer, for he wages unceasing warfare against them, and he has perfectly succeeded in emancipating himself from them as well as from common sense. In the choice of his illustrations and examples he has descended into the scums of society, and after gathering up all the filth possible, an unsavory mess is placed before us which smells rank to heaven, and which, introduced with long-winded apologies, he demands of us to accept, as a true representation of the present state of society. In order to catch the general reader, the whole work is infiltrated with moral sentences which spout forth as freely as the tears of the professional mendicant, and are as genuine as the protestations of innocence on the part of a convicted thief. The binding and printing is excellent, but if the very worst of material had been used, we would consider it too good for printing a book which is an outrage upon common decency and an insult to common sense. But the cloven foot cannot be concealed. While groaning over this wicked world, and shedding crocodile tears, the writer smacks his lips at, and rolls each separate piece of filth of this vile compound under his tongue as if it were a delicious morsel. The anonymous scribifax threatens to inflict another book upon society. We suggest, 1, a decent subject; 2, a decent title, and 3, a decent writer, who is not ashamed to publish his name on the title page."—*Physic and Pharmaceutist.*

**Recent Deaths.**

Prof. Geo. C. Blackman, M.D., Cincinnati.

E. M. Clark, M.D., Detroit.

Died, near Quincy, Ill., July 12th, 1871, of phthisis pulmonalis, Augustus E. Roux, æt. 25, student of Rush Medical College, Chicago, Ill., class of 1868–69.

"***The Student's Chart of the Sympathetic Nerve,***" by RALPH M. TOWNSEND, M D., Assistant Demonstrator of Anatomy in the Jefferson Medical College.

This is a convenient eight-by-ten map, useful to the student for reference, and, costing only a few cents, it is accessible to all. It represents the pre-vertebral ganglia, with their connecting cords; the ganglion of Ribes, ganglion impar, the hypogastric, aortic, solar, cardiac, facial, carotid and cavernous plexuses; the cephalic ganglia, branches of communication between the ganglia, with the cerebral and spinal nerves, and branches of distribution to the viscera, etc. This gives one a very good general idea of the distribution and relations of the different parts of the sympathetic system. The sympathetic nerves are colored yellow; the motor, blue; and the sensory, red.

Charles C. Chatfield & Co., of New Haven, have in press the last two sermons preached by President Woolsey, before retiring from the Presidency of Yale. One of them is his farewell sermon delivered July 2. This was pronounced, by those who heard it, to be the best he ever preached. The other is his last baccalaureate, delivered July 9. They will be printed in elegant style and bound up in paper (60 cents), and in cloth ($1.00).

